# Why cardio-rheumatology?

**DOI:** 10.1093/ehjcvp/pvag045

**Published:** 2026-06-26

**Authors:** Anne Grete Semb, Eirik Ikdahl, Anne Kerola

**Affiliations:** Department of Rheumatology, Division of Research and Innovation, Diakonhjemmet Hospital, Diakonveien 12, 0370 Oslo, Norway; Department of Rheumatology, Division of Research and Innovation, Diakonhjemmet Hospital, Diakonveien 12, 0370 Oslo, Norway; Department of Internal Medicine, Päijät-Häme Central Hospital, Wellbeing Services County of Päijät-Häme, Keskussairaalankatu 7, 15850 Lahti, Finland; Institute for Molecular Medicine Finland, HiLIFE, University of Helsinki, Tukholmankatu 8, 00250 Helsinki, Finland

Many chronic conditions elevate cardiovascular (CV) risk, from kidney disease to frailty. Creating a dedicated subspecialty for each would be impractical, so why does Cardio-Rheumatology merit its own field (*Figure 1*). As part of this discussion, the European Society of Cardiology (ESC) has published a FOCUS on Cardio-Rheumatology website (https://www.escardio.org/education/content-collections/focus/cardio-rheumatology/) including links to related literature and two webinars.

**Figure 1:**
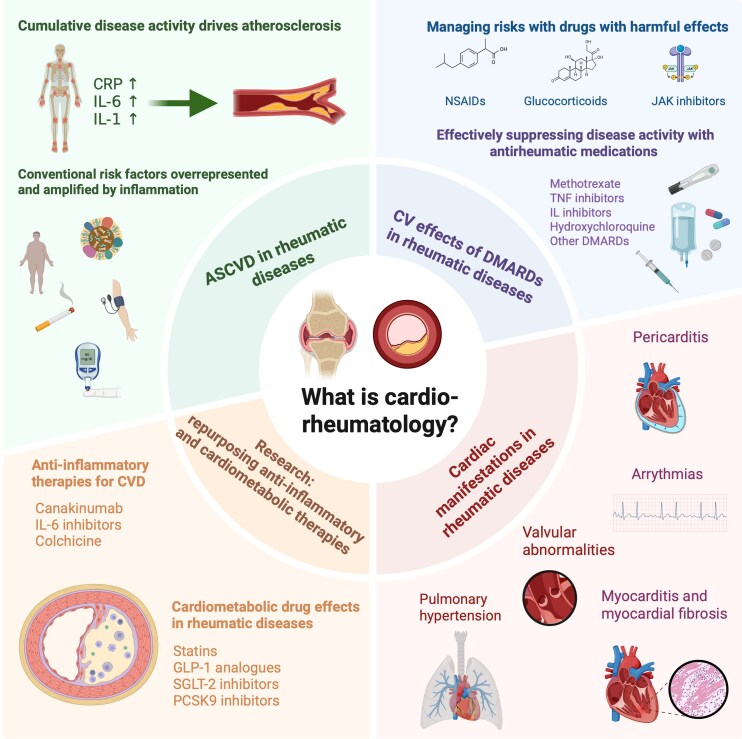
Key domains of cardio-rheumatology. Created in BioRender, Kerola (2026) https://BioRender.com/fsd1l76.

Nearly 15 million people in Europe live with inflammatory rheumatic diseases, ranging from inflammatory joint diseases like rheumatoid arthritis (RA) to connective tissue diseases such as systemic lupus erythematosus (SLE). These diseases may directly affect the CV system, manifesting as myocardial and pericardial inflammation, valvular abnormalities, arrhythmias, and pulmonary hypertension. Because risk stratification and clinical management depend heavily on the specific rheumatic phenotype and require careful consideration of anti-rheumatic therapies, close collaboration between cardiologists and rheumatologists is vital.

Furthermore, the risk of atherosclerotic CV disease remains elevated across these conditions despite advanced anti-rheumatic treatment.^1^ In certain diseases, this burden is comparable to that of diabetes mellitus as a CV risk equivalent. This reality particularly impacts women’s CV health. For instance, SLE has a striking 9:1 female-to-male predominance, and these patients face a fourfold increase in CV morbidity and mortality.

Large, international, multiethnic cohorts of patients with RA and SLE reveal a high prevalence of CV risk factors and inadequate risk factor control.^2,3^ This management gap is driven by several factors: a scarcity of evidence-based guidelines, underperformance of traditional risk prediction tools, unique risk factor profiles, and therapeutic inertia.

Cumulative inflammation burden drives a two to threefold increase in asymptomatic atherosclerotic CV disease in people with inflammatory rheumatic diseases. Moreover, standard CV risk calculators fail to capture the true disease burden. This underestimation is compounded by the «lipid paradox », where inflammation-induced lower lipid levels are tied to increased, rather than decreased, risk of myocardial infarction. Consequently, several ESC Guidelines recommend identifying subclinical atherosclerosis by carotid ultrasound in patients suspected to have very high CV risk, to ensure accurate reclassification into their appropriate risk classes.

Randomized controlled trials (RCTs) with hard CV endpoints in inflammatory rheumatic diseases remain scarce, leaving a gap in evidence-based guidelines. A notable exception is the TRACE-RA trial, an RCT comparing atorvastatin with placebo in primary prevention on major adverse cardiovascular events (MACE) in patients with RA, which demonstrated a non-significant 34% risk reduction.^4^ Despite lacking statistical significance, this magnitude of effect aligns with the MACE reductions established in landmark general population meta-analyses.^5^

Standard lipid-lowering and anti-hypertensive therapies are recommended as in the general population, offering comparable safety and tolerability. Importantly, baseline lipid levels, rheumatic disease activity, and concurrent anti-rheumatic medication do not alter the treatment intensity needed to reach recommended lipid targets. While anti-inflammatory medications, PCSK9 inhibitors, and GLP1 receptor agonists show promise for CV mitigation, further evidence is warranted specifically for people with inflammatory rheumatic diseases.

We advocate Cardio-Rheumatology as an essential collaboration at the interface of cardiology and rheumatology, uniting their complementary expertise to optimize CV prevention and management in this high-risk patient population.

## Data Availability

No new data were generated or analysed in support of this research.
